# Tetrasomy 8 Associated with a Poor Prognosis in Acute Monoblastic Leukemia: A Case Report

**DOI:** 10.7759/cureus.4068

**Published:** 2019-02-13

**Authors:** Fady Farag, Rewais Morcus, Preethi Ramachandran, Karan Josan, Jen Chin Wang

**Affiliations:** 1 Internal Medicine, Brookdale University Hospital and Medical Center, Brooklyn, USA; 2 Oncology, Brookdale University Hospital and Medical Center, Brooklyn, USA

**Keywords:** acute myeloid leukemia, tetrasomy 8, poor prognosis, karyotyping, aml

## Abstract

We report a case of a 47-year-old male from West Africa who presented with sepsis and was found to have acute monoblastic leukemia associated with tetrasomy 8 detected on bone marrow samples. This was the only chromosomal abnormality found. Tetrasomy 8 is a rare genetic finding that has been reported in acute myeloid leukemia (AML), predominantly the monocytic lineage. It carries a poor prognosis with a high mortality rate.

## Introduction

Trisomy 8 is one of the most encountered chromosomal aberrations found in acute myeloid leukemia (AML) and myelodysplastic syndrome [1]. However, tetrasomy 8 is a rare genetic finding with only 33 cases reported [2]. Tetrasomy 8 was linked to the monocytic lineage and was found to be the single genetic abnormality in about half the reported cases. It was found to carry poor prognosis with an aggressive course [3]. We report here a case of AML associated with tetrasomy 8 in a patient who presented with sepsis secondary to community-acquired pneumonia and malaria.

## Case presentation

**Figure 1:**
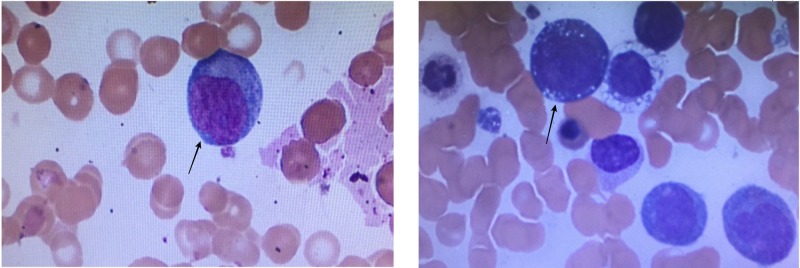
Peripheral Blood Smear Picture

We report a case of a 47-year-old male patient from Mali, West Africa, who presented with intermittent fever, chills, increased fatigue, decreased appetite, and diffuse back and abdominal pain for three weeks. He reported a 35-pound weight loss over three months. He had a history of malarial infection six months before, which was treated. He endorsed traveling through Africa until a recent illness. At the time of admission, his temperature was 102.3 F, blood pressure was 103-110/59-64 mmHg, and heart rate was 92-113 bpm, saturating at 99% on room air. A blood examination showed a hemoglobin level of 4.4, a mean corpuscular volume (MCV) of 87.5, a platelet count of 13, a white blood cells (WBC) count of 6.4, and an international normalized ratio (INR) of 1.73. Other values included serum sodium 132, lactate 4.6, alkaline phosphatase 133, total bilirubin 1.9, direct bilirubin 1.6, and albumin 2.8. The malarial screen was negative. A chest X-ray showed bilateral lower lobes infiltrates consistent with pneumonia. A computed tomography (CT) scan of the chest, abdomen, and pelvis showed mild bilateral areas of focal infiltrates and consolidation consistent with pneumonia and small right scrotal hydrocele. A peripheral blood smear (Figure 1) showed few monoblasts, few dysplastic features, and erythrocytes with intracellular inclusions. He received supportive care with blood and platelet transfusions. He was treated with intravenous ceftriaxone and intravenous azithromycin for community-acquired pneumonia and atovaquone for malaria.

**Figure 2:**
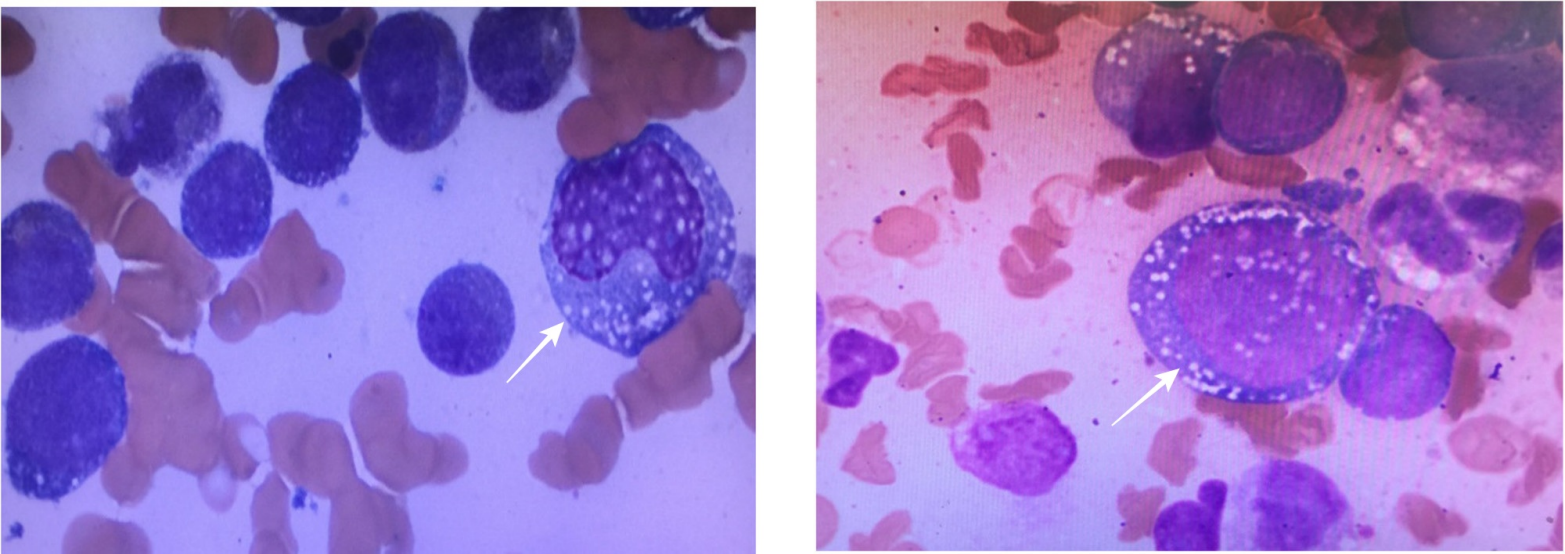
Bone Marrow Aspiration Showing Monoblasts Monoblasts are the largest blasts of all the hematopoeitic cell lines present in the bone marrow. They have a large, round, centrally-placed nucleus with soft, fine-stranded chromatin. They normally have a single, large, prominent nucleolus. The cytoplasm is very generous and has a fine, grainy texture.

A bone marrow aspirate was done (Figure 2), and the smear showed hypercellular marrow with monoblasts, erythroblasts, maturation defect, and few megakaryocytes. Further examination showed hypercellular marrow for age with sheets of immature mononuclear cells (blasts and monocytes) diffusely occupying marrow cavities. Table 1 shows the differential count of the bone marrow cells. Blasts comprised approximately 50% of marrow elements, confirmed by immunostaining with CD34 and CD117. Monocytes comprised approximately 20%-30% of marrow elements. The blasts were medium to large with increased nucleus-to-cytoplasm (N/C) ratio, fine chromatin, and prominent nucleoli.

Table 1 presents the bone marrow differential count.

**Table 1:** Bone Marrow Differential Count

Cell type	Results	Normal range
Myeloblasts	17%	0-3%
Promyelocytes	5%	2-8%
Myelocytes	9%	10-13%
Metamyelocytes	8%	10-15%
Neutrophils / Bands	15%	25-40%
Monocytes	22%	0-1%
Eosinophils	4%	1-3%
Basophils	1%	0-1%
Lymphocytes	8%	10-15%
Plasma Cells	1%	0-1%
Pronormoblasts	1%	0-2%
Normoblasts	9%	15-25%

These results were consistent with a diagnosis of acute myeloid leukemia (non-APL), best classified as acute myelomonocyte leukemia (AMML). Flow cytometry analysis from the aspirate showed blasts (12%-15%) that were positive for CD34 (partial), CD117, HLA-DR, CD13, CD33, and CD38. Monocytes (~25%) were positive for CD2, CD4, CD11b, CD11c, CD13, CD14, CD33, CD38, CD45, CD64, and HLA-DR. CD56 was negative.

**Figure 3:**
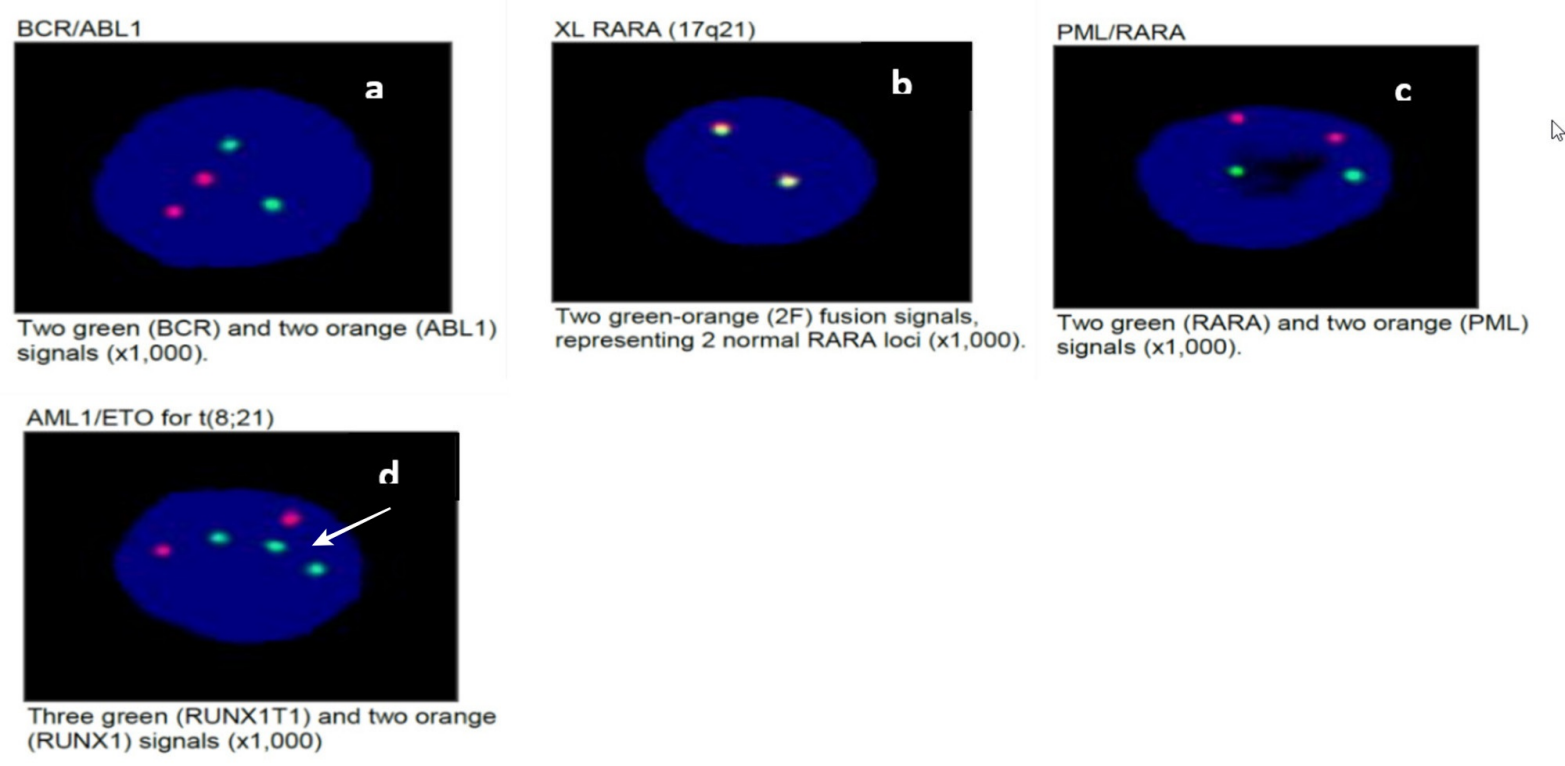
Fluorescence In Situ Hybridization Results Note in (d) three green dots representing three copies of RUNX1T1 and two orange dots representing RUNX1

CCAAT/enhancer-binding protein alpha (CEBPA) mutational analysis was not detected. A fluorescence in situ hybridization (FISH) test (Figure 3) showed no evidence of RARA rearrangement, no evidence of BCR/ABL rearrangement, no evidence of PML/RARA gene rearrangement, and no evidence for RUNX1/RUNX1T1 rearrangement; however, a subset of cells showed an abnormal hybridization pattern, consistent with gain of 8q or trisomy 8 (Figure 3D). There was no evidence of MLL gene locus 11q23 translocation and no evidence of CBFB [inversion (16) or translocation (16;16)] gene rearrangement.

**Figure 4:**
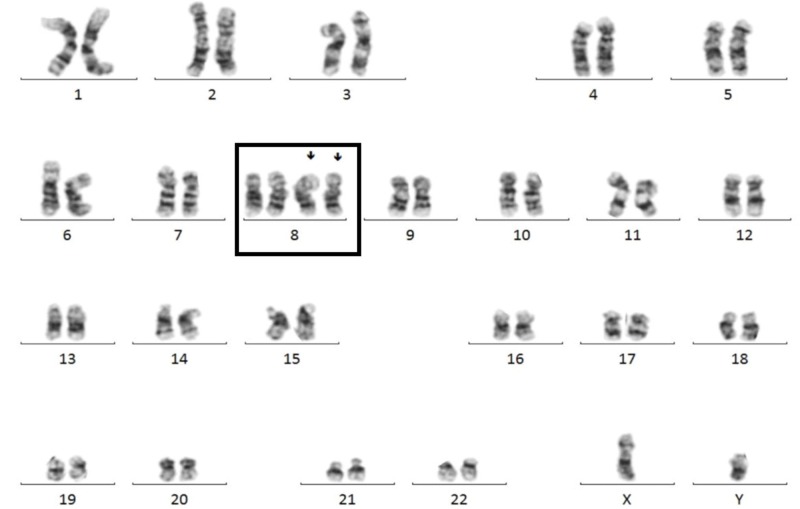
Karyotype of the Patient Showing Tetrasomy of Chromosome 8

OnkoSight ^TM^ (Bio-Reference Laboratories, Inc., NJ, USA) NGS AML panel sequencing identified a frameshift mutation in ASXL1 (p.Gly646Trpfs*12), a frameshift mutation in RUNX1 (p.Tyr281Leufs*319), and a hotspot missense mutation in DNMT3A (p.Arg882His). Cytogenetics revealed an abnormal male karyotype with tetrasomy of 8 (49, XY, 8+, 8+) (Figure 4).

## Discussion

Tetrasomy 8 is a rare event in hematologic disorders, associated with monocytic neoplasia (~50% of cases). Most frequently, it’s associated with AML-M5 [4]. Other conditions reported in association with isolated tetrasomy 8 include AML-M0, AML-M1, AML- M2, AML M7, MDS, and ALL [5]. Tetrasomy 8, when present, offers a major proliferative advantage and an aggressive course with overall median survival of six to seven months even with intensive therapy [4].

Tsirigotis et al. reported a case of a 25-year-old woman with AML-M5 associated with tetrasomy 8 as the sole chromosomal abnormality [2]. She received induction chemotherapy with cytarabine, idarubicin, and etoposide (7+3+3) after which she achieved complete remission but then relapsed in three months. Yan et al. reported two cases, the first one with AML-M4 with tetrasomy 8 as the sole chromosomal abnormality [4]. He received cytarabine and idarubicin but developed systemic mycosis and died on Day 25. The second patient was diagnosed with AML-M2 with two abnormalities. In the first clone, a Ph chromosome was seen as the sole chromosome abnormality, and in the second clone, tetrasomy 8 was detected in association with additional abnormalities including double Ph chromosomes, trisomy 18, and disomy Y. He was treated with cytarabine and idarubicin and entered into remission. However, he relapsed in three months. He was treated again but he didn’t respond and died nine weeks later of an aspergillus lung infection. Aktas et al. reported another case of a 14-year-old girl with AML-M7 with tetrasomy 8 and trisomy 6, 9 and 12 as secondary changes. She initially achieved remission but relapsed in five months and died of intracranial bleeding [6].

Tetrasomy 8 can occur by either of two mechanisms: (a) two consecutive events of single non-disjunction of chromosome 8 or (b) a single event of double non-disjunction of chromosome 8 [2]. No metaphases with trisomy 8 were observed in our patient. The absence of trisomy 8 might be a result of the longer mitotic duration of tetrasomic cells or of the higher proliferative advantage of tetrasomic cells compared to trisomic cells [7]. It is not clear what genes on chromosome 8 play a role in the pathogenesis of this disease or how it may affect the relapse [8]. However, some genes that might be involved in leukemogenesis on chromosome 8 are the following: MYC in 8q24, MOS in 8q22, and RUNX1T1 [9, 10]. These should be considered as potential causes of malignant transformation but further studies are required to prove this and the mechanisms involved with the malignant transformation [11].

## Conclusions

In this report we shared our findings of an unusual phenomenon and how we reached the diagnosis. What was first perceived as a simple case of sepsis turned out to be one of the rarest aberrancies in myeloid disorders. Tetrasomy 8 is a rare genetic abnormality in hematologic disorders including acute myelogenous leukemia. It is an independent poor prognostic marker in patients with acute myelogenous leukemia. The ongoing improvements in molecular and cytogenetic approaches will provide further information on the exact role of tetrasomy 8 in leukemogenesis.
